# Primary bovine embryonic fibroblasts demonstrate variable fitness following infection with highly pathogenic avian influenza H5N1 strains and are susceptible to a recently circulating human 2009 pandemic lineage H1N1 strain

**DOI:** 10.1128/spectrum.03285-25

**Published:** 2026-02-23

**Authors:** Grace K. Wenger, Deann T. Snyder, Justin R. Prigge, Allyson H. Turner, Sara A. Jaffrani, Edward E. Schmidt, Emily A. Bruce, Emma K. Loveday

**Affiliations:** 1 Microbiology and Cell Biology Department, Montana State University 33052, Bozeman, Montana, USA; 2 Department of Microbiology and Molecular Genetics, University of Vermont 169979, Burlington, Vermont, USA; 3 Redox Biology Laboratory, University of Veterinary Medicine 72408, Budapest, Hungary; Universiteit Utrecht, Utrecht, the Netherlands

**Keywords:** viral pathogenesis, cattle, influenza, virulence, avian viruses

## Abstract

The recent emergence of highly pathogenic avian influenza (HPAI) H5N1 (clade 2.3.4.4b, genotype B3.13) in dairy cattle presents substantial challenges to the agricultural sector and public health. Mechanistic studies of infection and transmission in cattle have proven difficult due to animal handling restrictions and the limited availability of established cell culture models. Primary bovine embryonic fibroblasts (BeEFs) were isolated and investigated here as a model to study influenza A virus (IAV) infection dynamics. We compared sialylation profiles, infectious virus production, viral replication, and plaque morphology in BeEFs following infection with the bovine HPAI H5N1 and an earlier 2.3.4.4b genotype (B1.1) isolated in 2022. The data presented here demonstrate increased expression of α-2,3 sialic acids compared to α-2,6 sialic acids in BeEFs, similar to sialylation profiles previously reported in bovine mammary tissue. These data also display increased viral fitness of the bovine origin HPAI H5N1 strains across bovine and avian cell lines, consistent with previous characterization in bovine mammary tissue. Furthermore, BeEFs were fully susceptible to a 2022 H1N1pdm09-like IAV strain while maintaining resistance to the 2009 H1N1pdm09 IAV as previously characterized in mammary cells. This study highlights the ongoing zoonotic adaptation of HPAI H5N1 in mammals and the potential for coinfection with select human H1N1 2009 pandemic lineage strains, enabling the potential development of reassortant strains. These data support the ability of BeEFs to serve as a complementary *in vitro* system for studying IAV infections in bovine hosts.

**IMPORTANCE:**

Zoonotic spillover to humans with avian influenza A subtypes, such as H5N1, can have extraordinarily high mortality rates. Recently, highly pathogenic avian influenza (HPAI) H5N1 has spread to dairy cattle and caused widespread disease in over a thousand herds across the United States. This widespread infection not only poses notable economic challenges to agricultural industries but also represents a notable concern to human public health. While studies of infection dynamics of HPAI H5N1 in cattle remain crucial, animal handling restrictions and a lack of well-characterized cell culture models make this work challenging. The significance of our research lies in identifying an *in vitro* system—primary bovine embryonic fibroblasts (BeEFs)—as a physiologically relevant *in vitro* system for studying these infection dynamics, helping to mitigate the limitations imposed by stringent animal handling requirements.

## OBSERVATION

Since March 2024, highly pathogenic avian influenza (HPAI) H5N1 (clade 2.3.4.4b) has been identified in 1,080 U.S. dairy cattle herds across 18 states, resulting in reduced milk production and mastitis among infected animals (1–3). This widespread transmission poses serious economic threats to agriculture, as control measures and biosecurity enhancements can cause serious financial burdens (4, 5). Infected bovine mammary glands release high concentrations of virus into milk, posing an additional risk of infection to humans exposed to unpasteurized milk (1). Since March 2024, 41 human cases from exposure to infected cattle have been documented (1, 2, 6–8). However, in-depth studies of HPAI H5N1 within bovine hosts are difficult due to animal handling restrictions and a lack of well-characterized primary *in vitro* models.

To improve understanding of HPAI H5N1 infection dynamics in cattle, we acquired primary bovine embryonic fibroblasts (BeEFs) from a mixed breed cow. We determined their susceptibility and permissivity compared to DF-1 cells, a chicken fibroblast cell line, to HPAI H5N1 viruses from multiple clades. We investigated sialic acid profiles and susceptibility of BeEFs to human 2009 pandemic-lineage influenza A virus (IAV) and HPAI H5N1 strains, which demonstrated consistency with previous characterizations of mammary tissue (9–12). Our results show that newly circulating bovine B3.13 genotype H5N1 virus demonstrates increased fitness compared to the avian B1.1 genotype H5N1 virus from prior to the bovine H5N1 outbreak, and that cattle have potential capacity to operate as mixing vessels for influenza A strains.

### Characterizing BeEF and DF-1 cells following HPAI H5N1 infection

**Fig 1:**
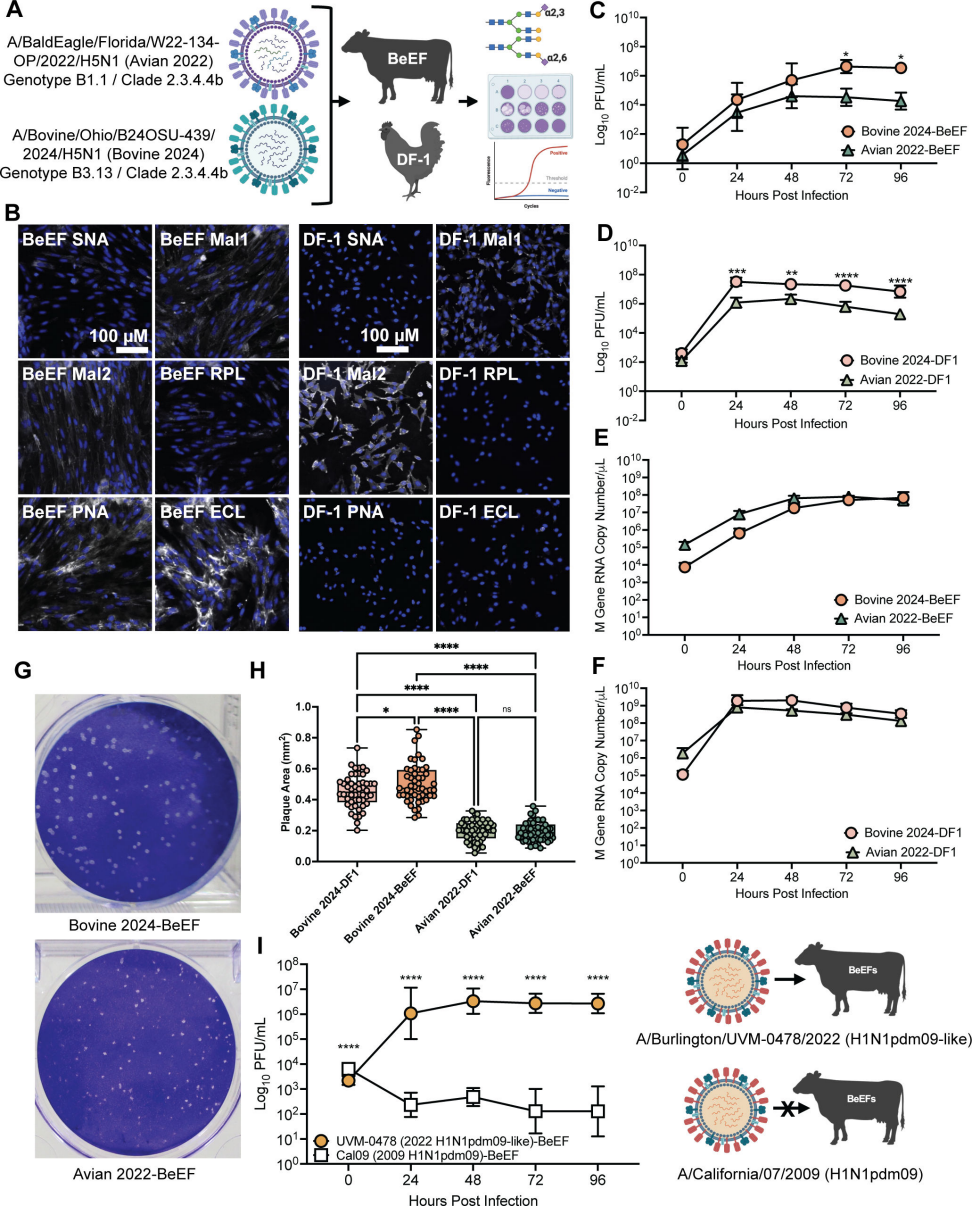
Characterizing BeEF and DF-1 cells. (**A**) A schematic of the experimental design: infection of BeEF and DF-1 cells with H5N1 isolated before (avian 2022 H5N1) and during (bovine 2024 H5N1) the bovine HPAI outbreak. Cells were analyzed for the presence of different sialic acid receptors, infectious virus production, and viral replication. (**B**) Sialylation profiling displaying lectin-binding (white/gray) of α-2,3- and α-2,6-specific lectins to BeEF and DF-1 cells and DAPI staining of nuclei (blue). MAL1 (*Maackia amurensis* lectin I), MAL2 (*Maackia amurensis* lectin II), and RPL are specific to α-2,3 receptors, and SNA (*Sambucus nigra* lectin) is specific to α-2,6 receptors. Representative images of each lectin are shown at the same magnification. Scale bar = 100 µm. Growth curves (MOI 0.1) of avian 2022 and bovine 2024 H5N1 on BeEFs (**C**) and DF-1s (**D**). Data represent a minimum of two independent replicates analyzed by two-way ANOVA. Viral replication as measured by RT-qPCR for the viral M gene in total RNA isolated from BeEFs (**E**) and DF-1s (**F**). Data represent a minimum of two independent replicates analyzed by two-way ANOVA. (**G**) Representative images of plaques formed by avian 2022 and bovine 2024 H5N1 infection of BeEFs. (**H**) Comparison of plaque areas reveals significantly reduced plaque sizes for avian 2022 H5N1 in both BeEFs and DF-1 compared to bovine 2024 H5N1. A minimum of 50 plaques were analyzed for each virus and analyzed by one-way ANOVA. (**I**) Growth curve of Cal09 H1N1pdm09 and 2022 UVM-0478 H1N1pdm09-like strains on BeEFs. Data represent a minimum of three independent replicates analyzed by two-way ANOVA. *P < 0.05, **P < 0.01, ***P < 0.001, ****P < 0.0001.

To determine whether BeEFs are susceptible to A/bovine/Ohio/B24OSU-439/2024 H5N1 and A/bald eagle/Florida/W22-134-OP/2022 H5N1 HPAI H5N1 and can function as a potential model system for studying HPAI infection dynamics in cattle, we performed a comparative study of BeEFs and DF-1s infected with an avian B1.1 genotype H5N1 isolated prior to the bovine outbreak and bovine B3.13 genotype H5N1 isolated at the beginning of the bovine outbreak. (Fig. 1A). Viral supernatant growth curves and total M gene in RNA from BeEFs and DF-1s infected with the different HPAI H5N1 strains were performed to determine fitness of each virus strain. Sialylation profiling was utilized to determine the presence of avian (α-2,3) and mammalian (α-2,6) sialic acid receptors to better understand cell susceptibility to strains isolated from various host species.

### Avian-origin IAV receptors are present on BeEF cells

Characterizing receptor prevalence across different cell types provides critical insight into IAV infection dynamics in multiple species. We compared fluorescence images of BeEFs and DF-1s (Fig. 1B) exposed to α-2,6- (SNA) and α-2,3- (MAL1, MAL2, and RPL-Sia1) specific lectins. MAL1 and MAL2 demonstrated clear binding to BeEFs, while SNA demonstrated faint binding to BeEFs. Overall, α-2,3-specific lectins demonstrated increased binding to BeEFs compared to α-2,6-specific lectins. Similarly, MAL1 and MAL2 lectins demonstrated clear binding to DF-1s, while SNA demonstrated limited to no binding. In contrast to BeEFs, binding of PNA and ECL to DF1 cells was minimal. Overall, α-2,3-specific lectins demonstrated increased binding to DF-1s compared to α-2,6-specific lectins.

### Bovine-origin H5N1 shows increased fitness in bovine and chicken primary cells

To assess potential differences in viral replication between the 2022 avian and 2024 bovine H5N1 strains, growth curves were performed following a multiplicity of infection (MOI) of 0.1 in BeEFs (Fig. 1C). These results showed a significant increase in bovine H5N1 virus production at multiple time points compared to avian H5N1. Growth curves were also performed in DF-1s, a cell type in which bovine-origin H5N1 has been previously characterized (13), with similar results (Fig. 1D). Interestingly, both the avian and bovine H5N1 strains produced higher titers overall in the DF-1s compared to BeEF cells. As the main site of infection in dairy cattle is the mammary gland, there may be additional host factors that are not present within our BeEFs that limit overall virus production compared to the DF-1 cells.

In addition to growth curves, we quantified the amount of viral RNA inside infected host cells. Following infection, host cells were lysed, and the levels of viral RNA were quantified via qRT-PCR. Viral M gene levels were analyzed and revealed efficient viral infection and replication with no significant difference between the two virus strains in both the BeEFs (Fig. 1E) and DF-1s (Fig. 1F). This contrasted with the differences seen in infectious virus produced and suggests that, while both viruses can produce similar levels of viral RNA, bovine H5N1 is able to more efficiently produce infectious virus in both BeEFs and DF-1 cells.

To further evaluate potential fitness differences, we analyzed plaque size from virus collected from BeEFs and DF-1 cells infected with bovine or avian H5N1 and titered on MDCK-SIAT1-TMPRSS2 (MST) cells, with representative images shown in Fig. 1G. Plaques from BeEFs and DF-1s infected with bovine H5N1 were significantly larger than plaques from avian H5N1-infected cells, demonstrating increased fitness of the bovine H5N1 strain across both species models (Fig. 1H) (14–17). Interestingly, the plaques produced from bovine H5N1-infected BeEFs were significantly larger than those from bovine H5N1-infected DF-1s, further highlighting the increased viral fitness of the bovine-origin virus in our bovine cell model.

### Bovine primary cells are susceptible to recently circulating human seasonal IAV

Previous infections of bovine mammary cells with the IAV PR8 strain found that virus could be secreted in the milk for multiple days (18). In addition, seropositivity and isolation of human and human-like IAVs from natural infection in cattle have previously been reported (19). Therefore, we investigated the susceptibility of BeEFs to circulating human IAV strains. BeEFs were infected with the 2009 pandemic strain, A/California/07/2009 (Cal09 H1N1pdm09), and a more recent pandemic lineage strain isolated from an IAV-positive clinical specimen, A/Burlington/UVM-0478/2022 (UVM-0478 H1N1pdm09-like) (20). Viral replication was measured via plaque assays following low-MOI growth curves (Fig. 1I). The growth curves revealed that BeEFs are not susceptible to the original Cal09 H1N1pdm09, with limited detectable plaques over 96 h post-infection (hpi), most likely representing remaining inoculum. Notably, BeEFs were fully susceptible and permissive to the 2022 UVM-0478 H1N1pdm09-like strain, as we observed a multi-log increase in infectious virus released at 24 hpi that was maintained through 96 hpi (Fig. 1I). These results are particularly interesting as they suggest significant variation from the original Cal09 pandemic lineage to the 2022 UVM-0478 H1N1pdm09-like strain that allows for efficient infection and replication within BeEFs.

The emergence of HPAI H5N1 2.3.4.4b strains across a variety of hosts poses a significant threat to both the agricultural industry and human health. While zoonotic spillover events into humans are rare due to restrictions that limit viral replication, human infection with bovine origin H5N1 has occurred multiple times over the past year (6, 7, 21, 22). Continued adaptation and evolution of HPAI H5N1 2.3.4.4b strains within avian, bovine, and human hosts indicate the virus’s continued zoonotic potential, increasing pandemic potential (22–24). However, there is a lack of well-described *in vitro* bovine models to study IAV. To address this need, we infected BeEFs, which are primary, non-immortalized fibroblasts that retain native sialic acid profiles and intact antiviral responses, with multiple IAV strains (25, 26). Our results are similar to recently published work on bovine mammary tissue (9–11). We compared HPAI H5N1 infection dynamics in primary BeEFs to chicken DF-1 cells to determine their capacity to support infection with bovine-origin and avian-origin HPAI H5N1. Similar to recent studies in bovine mammary cells, BeEFs support higher replication of bovine-origin HPAI strains compared to earlier clade avian-origin viruses (9, 10). Furthermore, we have demonstrated sustained infection of recently circulating (2022 UVM-048 H1N1pdm09-like) human-origin IAV in BeEFs, although they remain refractory to infection with the precursor 2009 pandemic H1N1 strain. Previous research demonstrated slight permissivity of bovine mammary cells to a human H3N2 virus isolated in 2013, suggesting that evolution of human IAV strains may have expanded their capacity to infect bovine hosts (9, 10). In addition, we found that sialic acid expression in BeEFs was consistent with previous reports on bovine mammary tissue further validating BeEFs as a model for studying influenza A virus within a bovine host (9–12). Taken together, our findings underscore the value of BeEFs as a complementary *in vitro* system for studying host-specific adaptations of influenza A viruses in cattle and advancing our understanding of cross-species transmission dynamics (9–12, 27–29).

## SUPPLEMENTAL MATERIAL

Supplemental material spectrum.03285-25-s0001.pdf Supplemental methods.

ASM does not own the copyrights to Supplemental Material that may be linked to, or accessed through, an article. The authors have granted ASM a non-exclusive, world-wide license to publish the Supplemental Material files. Please contact the corresponding author directly for reuse.
