# Supplementary material for: Primary bovine embryonic fibroblasts demonstrate variable fitness following infection with highly pathogenic avian influenza H5N1 strains and are susceptible to a recently circulating human 2009 pandemic lineage H1N1 strain
Source: Microbiol Spectr. 2026 Feb 23;14(4):e03285-25. doi: 10.1128/spectrum.03285-25 (PMC13055317; doi:10.1128/spectrum.03285-25)
Supplement: Supplemental material — Supplemental methods. [file spectrum.03285-25-s0001.pdf]

Supplementary Materials and Methods

Cells lines and maintenance

Madin-Darby canine kidney (MDCK) cells were obtained from Val Le Sage at the University of Pittsburgh and MDCK-SIAT1-TMPRSS2 (MST) cells were obtained from Jesse Bloom at the Fred Hutch Cancer Center (S1). Chicken embryonic fibroblast (DF-1) cells were obtained from Steve Baker at Lovelace Biomedical. Bovine embryonic fibroblasts (BeEFs) were provided by Ed Schmidt at Montana State University. All cells grown were maintained in DMEM (Corning) supplemented with verified BVDV-free 10% FBS (Gibco), 1X penicillin-streptomycin (Gibco). MSU cell lines were grown with 2.5 μg/ml plasmocin (Invivogen) while UVM cell lines were grown without plasmocin. All cells were passed weekly or as needed. The BeEF cells are available upon request.

Harvesting BeEFs

Bovine fetal harvest procedures were reviewed and approved by the MSU IACUC, protocol # 1999-13. To favor high heterozygosity, an “outbred Montana free-range heifer” was purchased at public auction (Bozeman, Montana, Autumn, 1999), verified clear, cycled, and artificially inseminated with bovine semen from a local generic undocumented source. At 45 days post insemination (23 February 2020), a single early fetus in intact decidua was harvested by manual palpation into a liter of ice-cold PBS containing 2X antibiotic cocktail (“PBS-ab”, Gibco # 15240096). The full decidua was transported to the laboratory in PBS-ab on ice, transferred to a new beaker of PBS-ab, and the maternal decidual tissues were carefully dissected away down to the fetus-containing amniotic sac, which was maintained unbreached. This was surface-disinfected by 70% ethanol-dip for 30 sec and then dipped into a fresh beaker with sterile PBS-ab (to remove ethanol) in a biosafety cabinet and transferred into a sterile culture dish. The amniotic sac was breached and the fetus (~ 2 cm long) was transferred to a fresh dish. The dermis was gently collected and transferred into a 50 ml tube containing 10 ml 2X trypsin solution (Gibco # 15090046). After 20 min 37°C, 35 ml of DMEM + 10% newborn calf serum (NCS) + 1X antibiotics was added to quench the trypsin. Cells were sedimented by centrifugation (300 x g 10 min), supernatant was aspirated, and cells were dispersed by gentle trituration with 20 ml of DMEM + 10% NCS + 1X antibiotics and plated onto 10 x 10-cm cell culture dishes in the same medium. Media was replaced the next day. One day later, the cells had reached near-confluence; they were collected with trypsin, frozen as “P0” stocks, and maintained in liquid nitrogen thereafter. Diploid bovine karyotype (n = 60 chr) was verified through manual counting of metaphase chromosome spreads.

Influenza strains and production

The following strains of avian influenza viruses were obtained from Richard Webby (St Jude’s, Memphis, TN): A/bovine/Ohio/B24OSU-439/2024 H5N1 and A/bald eagle/Florida/W22-134-OP/2022 H5N1. A/Burlington/UVM-0478/2022 (H1N1pdm09-like) (UVM-0478) was isolated from an IAV positive clinical

specimen graciously provided by Dr. Jessica Crothers at the University of Vermont, the use of deidentified positive clinical specimens was approved by the University of Vermont Institutional Review Board (STUDY00000881) under a waiver of consent (20). A/California/07/2009 (Cal09) H1N1pdm09 was obtained from Chris Brooke at the University of Illinois at Urbana-Champaign. HPAI H5N1 viral stocks were generated from MDCK cells in infection media containing DMEM (Corning) supplemented with 0.3% BSA (MP Biomedicals), 1X penicillin-streptomycin (Gibco), 1mM HEPES (Gibco). For the H1N1 viruses, 2ug/TPCK-trypsin (Worthington Biomedical) was also added to the infection media. Supernatants were collected and centrifuged at 3,000 g for 5 min to remove cellular debris. The clarified viral supernatant was titered by plaque assay on MST cells and sequence verified then was used for all subsequent infections. All experiments with H5N1 strains were performed in the Jutila Research Biosafety Level 3 Lab at Montana State University, Bozeman, Montana. Human IAV stocks were produced as previously described (S2). Briefly, viral stocks were grown on MSTs in a T150 flask (Corning) and infected at an MOI of 0.001 in infection media supplemented with 1 ug/ml TPCK trypsin (Sigma-Aldrich) and incubated at 37°C until ~50% CPE was observed.

BeEF and DF-1 cell infections

Cells were counted the day of infection followed by inoculation at an MOI of 0.1. TPCK-Trypsin was added to infection media at 1 µg/ml for H1N1 infections but omitted for H5N1 strains. Cells were washed twice with PBS then inoculated for one hour at 37C. Following infection, inoculum was removed and replaced with fresh infection media. To collect the 0 hour time point, the fresh media was added for 10 minutes prior to collecting. The cells were returned to 37°C and supernatants were collected from individual wells every 24 hours for the duration of the experiment (96 hours).

Quantification of viral titers by plaque assay

HPAI H5N1 titers were determined via plaque assays with MST cells, as previously described (S3). Briefly, MST cells were cultured in complete DMEM, seeded onto 6-well plates at 7.5×10^5 cells per well and grown to confluence overnight. They were then incubated with serially diluted viral inoculum in 200 µl infection media for 1 h. Following inoculation, cells were over-layered with 1.5% carboxymethylcellulose, DMEM, 0.2mg/ml DEAE-dextran, 0.3% BSA, 1X penicillin-streptomycin, 1mM HEPES and 3.7g/L sodium bicarbonate and incubated for 3–4 days. Cells were then fixed with 4% paraformaldehyde for 30 minutes and stained with 0.1% crystal violet in water for 15-20 minutes. Plaques were counted and the titer was calculated based on the volume of inoculum plated and dilution counted.

Human H1N1 titers were performed as previously described (20). Briefly, MST cells were seeded at 1×10^6 cells per well of a 6 well plate and inoculated with serial ten-fold dilutions of each sample in DMEM. After one hour, cells were overlayed with 2 ml of a warmed 1:1 mixture of 2.4% Avicel RC-591 NF (Dupont) + 1X DMEM (Corning) + 1 µg/ml TPCK trypsin (Sigma-Aldrich). Cells were returned to 37C for 48 hours, and care was taken not to disturb the plates during this period. Finally, cells were washed with PBS (Corning), fixed with 4% formaldehyde (Honeywell) for 20 minutes and stained with 0.1% crystal

violet (Fisher) for five minutes. Plates were rinsed three times with water and allowed to dry before plaques were counted to determine viral titer.

Replicate titer values at each time point and condition were compiled and used to generate viral growth curves. Statistical comparisons of viral replication across timepoints and conditions were analyzed with a two-way ANOVA. Significance was defined as $p < 0.05$. Statistical analyses were completed using Prism.

M gene abundance in total RNA by RT-qPCR

RNA was extracted from cell lysates using the Invitrogen PureLink RNA mini kit (Invitrogen). RNA copy number of the IAV matrix protein gene (M gene) was determined using a standard curve and TaqMan RT-qPCR assay using previously described standards (S3, S4). The amplification primer sequence was as follows: M gene forward, 59-GACCRATCCTGTCACCTCTGAC-39, and M gene reverse, 59-AGGGCATTCTGGACAAATCGTCTA-39. The sequence of the M gene TaqMan probe was 59-/FAM/TGCAGTCCTCGCTCACTGGGCACG/BHQ1/-39. Working stocks of the primers and probe (Eurofins Operon) were prepared at 25 mM and 10mM, respectively, for use in the RT-qPCR. Samples were amplified using a SuperScript III Platinum one-step RT-qPCR kit (Invitrogen) with a final reaction volume of 25 µL Each reaction mix contained 400 nM M gene forward and reverse primers, 200 nM M gene TaqMan probe, 0.05 mM ROX reference dye, 5U/mL Superase RNase inhibitor (Invitrogen), and 5 µL of RNA template. Thermocycling was performed in an RT-qPCR machine (QuantStudio 3; Applied Biosystems) with the following cycling conditions: 1 cycle for 30 min at 60°C, 1 cycle for 2 min at 95°C, and 40 cycles between 15 s at 95°C and 1 min at 60°C.

Plaque Size Quantification

Images of plaques from A/Bovine/Ohio/B24OSU-439/2024/H5N1 and A/Bald Eagle/Florida/W22-134/2022/H5N1 infections of BeEF and DF-1 cells were captured following infection and staining on a light tracing box with built in scale (Amazon). Images were imported to Fiji (Image J) and the appropriate scale was calibrated using the included scale bar. For each virus-cell type combination, fifty distinct plaques were outlined manually and measured using the “Analyze Particle” function. The resulting measurements were compiled and analyzed using a one-way ANOVA. Significance was defined as $p < 0.05$. Statistical analyses were completed using Prism.

Sialylation Profiling by Lectin Staining

Chicken embryonic fibroblasts (DF-1) and bovine embryonic fibroblasts (BeEF) were seeded at 1×10^5 cells per well on an 8-well LabTek chambered coverslips (Thermo Fisher Scientific) and incubated for 24 hours until adherent. Cells were washed with PBS and then fixed using 4% PFA for 30 minutes at room temperature. To reduce non-specific binding, the cells were washed twice with PBS and incubated in a blocking solution of 2% BSA in PBS for 10 minutes at room temperature.

Lectin staining was performed using the Alpha 2,3/2,6 Sialylation Profiling Kit (ZBiotech) with modifications to adapt the manufacturer’s protocol to fixed adherent cells. Each of 6 wells per cell type

were stained with a distinct biotinylated lectin (100 µl) at a final concentration of 0.125 µg/100 µL in PBS and incubated at room temperature for 1 hour. After primary lectin incubation, cells were washed several times with PBS and then incubated with FITC-conjugated streptavidin (100 µl) at 7 µg/mL in PBS at room temperature for 1 hour. Cells were washed several times with PBS and then nuclei were stained with Hoescht solution (100 µl) at 2 µg/mL in PBS and incubated at room temperature for 10 minutes. Cells were washed several times and slides were stored in PBS until imaging.

Fluorescence microscopy was conducted using a 20X objective lens. Lookup table (LUT) settings were normalized across all images to enable direct comparison of the images. All staining experiments were repeated using independently plated cells with representative images shown. Additional images were acquired with adjusted LUT settings.

References

S1. Lee JM, Huddleston J, Doud MB, Hooper KA, Wu NC, Bedford T, Bloom JD. 2018. Deep mutational scanning of hemagglutinin helps predict evolutionary fates of human H3N2 influenza variants. Proc Natl Acad Sci USA 115:E8276–E8285. https://doi.org/10.1073/pnas.1806133115

S2. Dholakia V, Quantrill JL, Richardson S, Pankaew N, Brown MD, Yang J, Capelastegui F, Masonou T, Case K-M, Ajeian J, Woodall MNJ, Magill C, Freimanis G, McCarron A, Staller E, Sheppard CM, Brown IH, Murcia PR, Smith CM, Iqbal M, Digard P, Barclay WS, Pinto RM, Peacock TP, Goldhill DH. 2025. Polymerase mutations underlie early adaptation of H5N1 influenza virus to dairy cattle and other mammals. bioRxiv. https://doi.org/10.1101/2025.01.06.631435

S3. Loveday EK, Sanchez HS, Thomas MM, Chang CB. 2022. Single-cell infection of influenza A virus using drop-based microfluidics. Microbiol Spectr 10:e0099322. https://doi.org/10.1128/spectrum.00993-22

S4. Loveday EK, Zath GK, Bikos DA, Jay ZJ, Chang CB. 2021. Screening of additive formulations enables off-chip drop reverse transcription quantitative polymerase chain reaction of single influenza a virus genomes. Anal Chem 93:4365–4373. https://doi.org/10.1021/acs.analchem.0c03455
